# A randomized controlled study for the treatment of middle-aged and old-aged lumbar disc herniation by Shis spine balance manipulation combined with bone and muscle guidance

**DOI:** 10.1097/MD.0000000000023812

**Published:** 2020-12-18

**Authors:** Jinhai Xu, Xing Ding, Jinze Wu, Xiaoning Zhou, Kun Jin, Ming Yan, Junming Ma, Xuequn Wu, Jie Ye, Wen Mo

**Affiliations:** Department of Orthopaedics, Longhua Hospital, Shanghai University of Traditional Chinese Medicine, No. 725, Wanping South Road, Fenglin Street, Xuhui District, Shanghai, China.

**Keywords:** LDH, manipulation, ptotocol, therapy

## Abstract

Ninety percent of elderly patients with lumbar disc herniation (LDH) have problems with the mechanics of the spine and muscle tissue. Shi-style spine balance manipulation combined with guidance (Daoyin) of muscle and bone as an alternative therapy for LDH can tone the muscle groups around the spine and maintain optimal mechanical and static sagittal balance of the spine. This study will be performed to investigate the effect of a combination of Shi-style spine balance manipulation and Daoyin therapy on LDH in middle-aged and elderly patients. In this non-blinded, randomized controlled trial, 72 eligible patients will be randomly divided into a treatment group (Shi-style spine balance manipulation combined with Daoyin therapy) and a control group (lumbar mechanical traction). Before and after the intervention, lumbar X-ray and magnetic resonance imaging examinations will be performed to observe the sagittal balance parameters of the spine and pelvis and the lumbar muscle strength. The visual analog scale score, Oswestry disability index score, and pressure pain threshold will be evaluated at baseline and at 2, 4, 12, and 24 weeks. During the treatment period, any signs of acute adverse events, such as paralysis of the lower extremities or cauda equina syndrome, will be recorded at each visit. Although Shi-style spine manipulation combined with Daoyin therapy has been used in the treatment of LDH in middle-aged and elderly people in China for many years, there is no consensus on its effectiveness. This experiment will provide convincing evidence of the efficacy of Shi-style spine manipulation combined with Daoyin therapy in the treatment of LDH in middle-aged and elderly people.

## 1 Introduction

The main clinical symptom of LDH is waist and lumbocrural pain. Patients with severe LDH may even develop lower limb paralysis and incontinence.^[1]^ LDH has a high incidence and affects a wide range of people. It has become an important disease that affects peoples health, work ability, and quality of life.^[2]^ The risks associated with surgery are higher in elderly patients because they are more likely to have combined underlying morbidities such as hypertension and diabetes. LDH is characterized by a long disease course, high relapse rate, and difficulty obtaining good outcomes of radical treatment, resulting in long-term suffering. Therefore, the medical field faces a substantial challenge in establishing effective treatment and rehabilitation measures to reduce the incidence of recurrent attacks of LDH.

Research has shown that more than 90% of elderly and middle-aged patients with LDH have mechanical problems associated with their spinal and muscle tissues.^[3]^ Under normal circumstances, the “tendon” system composed of muscle groups and ligaments in the rear of the spine maintains the exogenous stability of the spine (i.e., its dynamic balance).^[4]^ The “bone” system composed of vertebral appendages and intervertebral discs maintains the endogenous stability of the spine (i.e., its static balance). The sagittal balance of the spine and pelvis is an important parameter when evaluating the biomechanical stability of the spine. “Tension band function” from erector spine muscle group of lumbar rear is the key to maintaining orthostatism and sagittal balance of the spine. An imbalance between the dynamic and static forces of the spine,^[5]^ which is associated with sagittal imbalance of the spine and pelvis along with atrophy and fat infiltration of the extensor muscles of the lumbar spine, greatly contributes to the occurrence and development of LDH.

In traditional Chinese medicine, manipulation is the main treatment method for LDH. Using massage to rotate the spine rotation, the protrusion of the nucleus pulposus and position of the nerve roots can be changed to remove adhesions, relieve compression, and expand the nerve root canal. In addition, such manipulation can promote repair of the posterior extensor muscles of the dorsolumbar spine^[6–8]^ by relieving muscle spasm, promoting muscle relaxation, dilating peripheral blood vessels, improving local anemia and anoxia, and eliminating tissue inflammation and edema. Through exercise and Daoyin (guidance) of the lumbar core muscles, the muscle strength of the posterior lumbar multifidus, erector spinae, and psoas major muscles can be enhanced; the coordination and flexibility of the spinal ligaments can be increased; muscle adhesion can be released; muscle elasticity can be restored; and muscle atrophy can be prevented.^[9,10]^ Such therapy plays an important role in maintaining or restoring the optimal balance of mechanical dynamic and static sagittal forces of the spine and pelvis, thus curing and preventing recurrence of LDH. Therefore, establishment of a standardized scheme of manipulation and guidance (Daoyin) in the treatment of LDH is of great clinical significance.

In one retrospective study, the sagittal balance parameters of the spine and pelvis as shown by lumbar vertebral magnetic resonance imaging (MRI) and X-ray examination were compared between 32 patients aged >50 years with LDH at L4/5 and L5/S1 and 32 asymptomatic healthy persons.^[11]^ The study showed that the degree of lumbar lordosis was smaller in patients with LDH (−43.52 ± 14.56 degrees in the LDH group and −48.3 ± 11.1 degrees in the normal group). The pelvic incidence (PI) was also smaller in patients with LDH (45.1 ± 9.6 degrees in the LDH group and 49.12 ± 10.02 degrees in the normal group). The sacral slope (SS) was smaller in patients with LDH (32.05 ± 9.40 degrees in the LDH group and 34.8 ± 7.8 degrees in the normal group), as was the pelvic tilt (PT) (10.3 ± 6.7 degrees in the LDH group and 17.53 ± 8.57 degrees in the normal group). The severity of thoracic kyphosis was greater in patients with LDH (27.6 ± 10.33 degrees in the LDH group and 24.2 ± 9.8 degrees in the normal group). At present, there is a lack of research on the parameters of spinal sagittal balance and lumbar bone structure in elderly patients with complicated LDH treated by spinal balance manipulation combined with Daoyin therapy. The above research results provide an important theoretical basis for the development of our current project, which is also a continuation of previous work. Pelvic traction is beneficial for alleviating the symptoms of nerve root compression and is a common method for treating LDH. There is no clear clinical evidence against the use of traction in the treatment of LDH. Therefore, patients in the control group of our proposed study will be mainly treated with pelvic traction.

### 1.1 Trial design

We herein describe the study protocol for an outcome assessor-blinded and data analyst-blinded prospective randomized controlled clinical trial.

## 2 Method/design

The purpose of this prospective randomized controlled clinical trial is to investigate whether Shi-style spine manipulation combined with Daoyin therapy can lead to improvements in middle-aged and elderly patients with LDH. The study will be conducted in the Department of Orthopaedics, Longhua Hospital, Shanghai University of Traditional Chinese Medicine. The principal investigator will be responsible for the entire project, including organization of the steering committee meetings. An independent steering committee will be responsible for participant safety, meetings, participant recruitment, and quality control. The coordination center will be responsible for communicating protocol modifications and providing materials. The trial will involve 4 weeks of treatment and 6 months of follow-up. After randomization, the patients will receive one course of treatment within 4 weeks. The results will be evaluated at baseline and at weeks 2, 4, 12, and 24.

### 2.1 Eligibility criteria

#### 2.1.1 Inclusion criteria^[12]^

Male or female participants who meet the following criteria will be eligible for the study:

- Provision of written informed consent
- Satisfaction of the diagnostic criteria for LDH
- Age of 40 to 75 years
- LDH duration of > 3 months
- Willingness to complete the required spinal balance manipulation treatment and Daoyin training on a voluntary basis

#### 2.1.2 Exclusion criteria

The exclusion criteria are as follows:

- Age of <40 or >75 years
- Past or current participation in other clinical investigations within the last 3 months
- History of failed nonsurgical treatment for 6 months with aggravation of symptoms
- Obvious decrease in muscle strength within a short period of time (muscle strength grade < 3) with symptoms of cauda equina syndrome
- Imaging diagnosis of complete intervertebral disc protrusion and prolapse
- History of severe lumbar trauma and lumbar surgery
- Pregnancy or preparation for pregnancy
- History of opioid analgesics, sedative hypnotics, or alcohol abuse
- Systemic diseases such as malignant tumors, diabetes, severe rheumatism, or severe osteoporosis
- Failure to understand or sign informed consent

**Figure 1:**
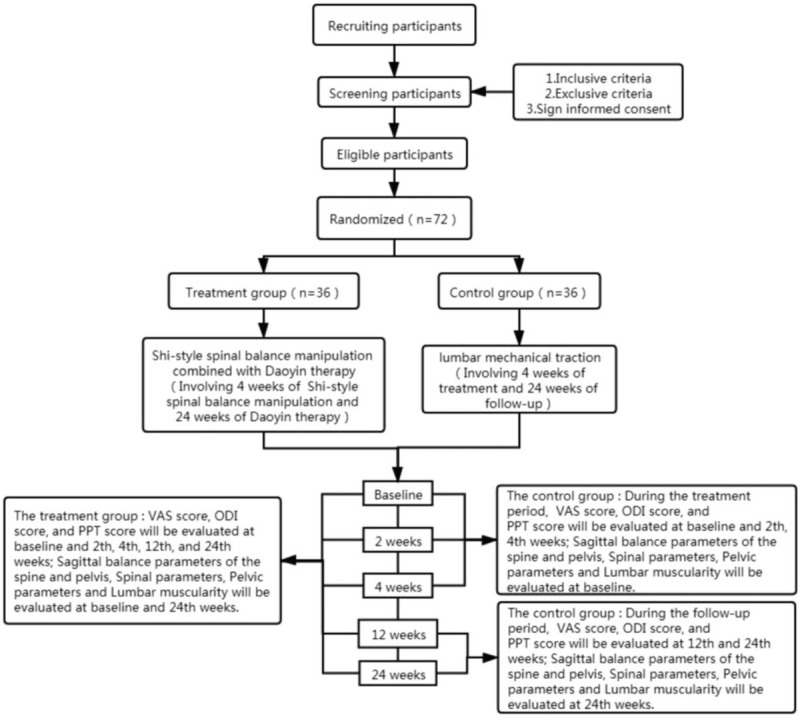
Consort flow diagram.

The individuals performing the participant recruitment, which is currently ongoing, were selected from the staff at Longhua Hospital Affiliated to Shanghai University of Traditional Chinese Medicine and its sub-centers. Prospective participants are being interviewed and informed of the eligibility criteria and procedures by the coordinator. Eligible participants are first be screened by a baseline assessment and then diagnosed based on their clinical presentation, physical examination findings, and imaging results. All patients are informed that participation in the trial is strictly voluntary and that they can withdraw from the trial at any time. If the patients choose to quit, the collected data will not be deleted; instead, it will be used for the final analysis. A compilation table containing all variables of interest and all potential risks will be completed by the research center. The information obtained will be stored in an electronic database for subsequent statistical analysis. Recruitment began in January 2020 and is expected to end in September 2021. The final follow-up of all participants will be completed by December 31, 2021. The participant processing and evaluation schedule is shown in Figure 1.

### 2.2 Ethics approval and consent to participate

This study will be conducted in accordance with the principles of the Declaration of Helsinki for Clinical Research.^[13]^ The trial protocol has been approved by the Research Ethics Committee of Longhua Hospital Affiliated to Shanghai University of Traditional Chinese Medicine (No. 2019LCSY015). All participants will be given sufficient time to make a decision to provide written informed consent prior to the study.^[14,15]^ The protocol has been registered in the Chinese Clinical Trial Registry (ID: ChiCTR1900021825).

### 2.3 Intervention

All patients will be treated by Shi-style lumbar manipulation combined with Daoyin therapy or mechanical lumbar traction. The patients in the treatment group will undergo Shi-style lumbar manipulation for 20 minutes every other day (3 times a week) for 4 weeks. They will also undergo Daoyin therapy of the muscle and bone once every other day for 6 months. If the patient has a visual analog scale (VAS) score of >7, analgesics may be allowed. All treatments and examinations will be free of charge to improve patients the compliance with the intervention plan. Analgesics will be allowed during the trial, but the patients will be required to record the medications taken.

### 2.4 Shi-style spinal balance manipulation

#### 2.4.1 Tendon soothing (Fig. 2a–c)

##### 2.4.1.1 Rubbing method

The palm surface is used to make a straight or circular rhythmic movement on the waist and back for 2 minutes.

**Figure 2:**
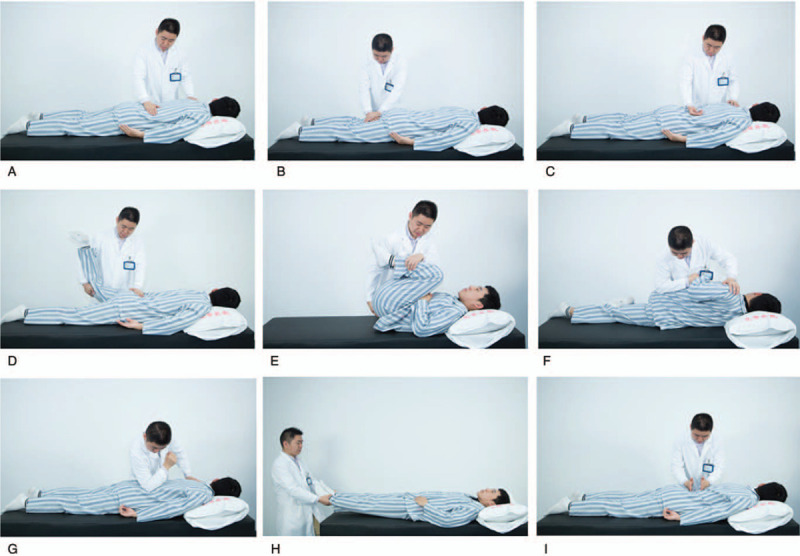
The Shi-style lumbar Balance manipulations include three steps and nine methods, the first step is Tendon soothing, Contains Rubbing, Kneading and Rolling methods (2a-c). The Second step is Osteopathic manipulation, Contains Extraction and extension, Lumbar flexion methods, Tilt (2d-f). The third step is Channel dredging, Contains Point, Shake, Pat methods (2g-i).

##### 2.4.1.2 Kneading method

With one hand or 2 hands overlapping, the root of the palm is placed on the patients waist and back, and the practitioner presses and kneads along the midline and sides of the spine, hips, thighs, and back of the calves for 2 minutes.

##### 2.4.1.3 Rolling method

The ulnar side of the palm (half-fist shape) is rolled on the patients waist and back, moving from the waist to the hips, thighs, and calf muscles for 2 minutes.

#### 2.4.2 Osteopathic manipulation (Fig. 2d–f)

##### 2.4.2.1 Extraction and extension

The patient is placed in the prone position. The physician stands on the left side of the patient, holding the patients knee joint with his or her right hand, holding the patients waist with his or her left hand, lifting the patients knee joint with his or her right hand, and pressing the waist vertically with his or her left hand; this process is repeated 3 to 5 times.

##### 2.4.2.2 Lumbar flexion

The patient is placed in the supine position with the hips and knees flexed and the front of the thighs touching the abdomen. The physician stands on the right side of the patient, holding the patients legs with his or her left hand and lifting the patients hips with his or her right hand, thus extremely flexing the lumbar spine; this process is repeated 3 to 5 times.

##### 2.4.2.3 Tilt

The patient lies on the right side first, with the right lower limb straight, the left lower limb flexed with hip and knee bent, the right hand straightened, the left hand flexed with the elbows on the waist, and the head lifted back later. The doctor stands on the right side of the patient, presses the patients left shoulder with his left hand and pushes backwards, and presses the right elbow to press the patients left iliac part and twists forward. Then let the patient turn over and repeat the above diagonal wrench method.

#### 2.4.3 Collateral dredging (Fig. 2g–i)

##### 2.4.3.1 Point method

The elbow joint is used to press the erector spinae muscles and sacroiliac muscles on both sides of the lumbar spine, and the thumbs are used to press the hips, thighs, posterior abdominal muscles, and calves. The pressure is held for 2 minutes.

##### 2.4.3.2 Shake method

The patients ankles are held with both hands, and small, continuous upward and downward shaking movements are made with slight force. This is continued for 2 minutes.

##### 2.4.3.3 Pat method

The palms or ulnar sides of the hands are used to pat the muscles on the back, hips, thighs, and back of the calf for 2 minutes.

### 2.5 Guidance (Daoyin) for muscle and bone

#### 2.5.1 *Leg lifts* (Fig. 3a1–4)

The patient lies on his or her back with the legs on both sides of the body or cross your hands in the back of your head. The left lower limb is lifted straight up to about 60° and maintained in this position for 5 seconds; it is then slowly lowered to the ground, and the process is repeated on the other side. Both lower limbs are then simultaneously lifted to about 30° and held in this position for 5 seconds; they are then slowly raised to 60° and held for 5 seconds. This process is repeated 5 times.

**Figure 3:**
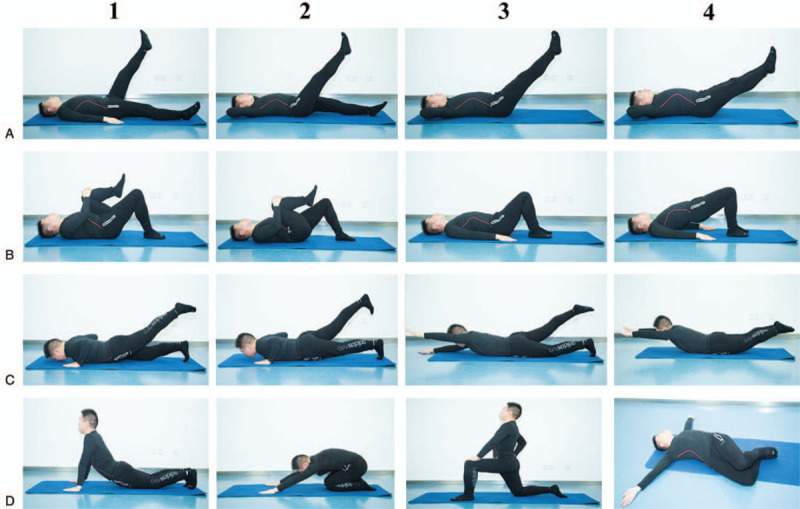
Guidance (Daoyin) for Muscle and Bone Contains four methods: Leg lifts (A1-4), Arch bridge (B1-4), Swallow exercise (C1-4), Stretching (D1-4).

#### 2.5.2 *Arch bridge* (Fig. 3b1–4)

The patient is placed in the supine position. He or she flexes the hips and bends the knees, embracing the left knee with both hands and moving the left knee close to the torso; this position is held for 5 seconds. The same process is repeated on the contralateral side for a total of 5 times. Next, the knee is flexed to 90°, bilateral heel and shoulder three-point support, as far as possible to straighten the buttocks and maintain for 5 seconds, a total of 5 groups of practice.

#### 2.5.3 Swallow exercise (Fig. 3c1–4)

The patient lies on his or her back with the elbows flexed on both sides of the body and the neck relaxed. The left lower limb is slowly lifted, keeping the thigh off the ground and maintaining this position for 3 seconds. The process is then repeated on the other side, and a total of 5 trials are performed. Extend the left hand with the head back and lift the upper body; this position is maintained for 3 seconds, and the process is repeated 5 times. Next, the hands are placed straight on both sides of the head, tilt back the head and lift the upper body while the legs are extended straight and lifted up; this position is maintained for 3 seconds and the process is repeated 5 times.

#### 2.5.4 *Stretching* (Fig. 3d1–4)

For abdominal stretching, the patient is placed in the prone position with the elbows flexed on both sides of the body in line with the shoulders, and the neck is relaxed. Both upper limbs are then slowly straightened, and this position is held for 15 seconds. For back stretching, abdomen sticking out of the front of thighs and hips touching heels for 15 seconds. For hip stretching, the patient kneels on 1 knee with the knee flexed to 90°, the thighs and body at a 90° angle, and the back straight; this position is held for 15 seconds. Finally, for waist stretching, the patient is placed in the supine position, the knee joint is flexed to 90° and turned to one side, keeping the shoulders and hips away from the ground. The patient then holds the outside of the thigh against the ground for 15 seconds.

### 2.6 Mechanical lumbar traction

Patients with pelvic traction lie supine on the traction bed, bend the hips and knees, place both feet on the bed, and keep waist attach to the bed. For the patient whose lumbar curvature uncurls or whose lumbar muscle is obviously tense, traction therapies should place a thin pillow approximately 1.5 cm high under his lower back of the waist. Traction posture is body bent at hips and knees. Traction weight is equal to 25% to 30% patients weight for the first time, 2 kg weight increased each time, until the traction weight reach at 40% to 60% of patients weight. The specific standard should be based on patients tolerance. Therapist uses intermittent traction method, lasts for 40 seconds, pauses for 10 seconds, 20 minutes each time, once every other day, 3 times a week, 4 weeks in all.

### 2.7 Outcome measures

#### 2.7.1 Primary outcome measures

Because pain is the most common symptom in patients with LDH, we chose the VAS score as one of the primary outcome measures for evaluating low back and leg pain. The VAS is a reliable and effective pain measurement tool. It is a 100-mm horizontal line showing “no pain” on the left (score of 0) and “ most severe pain possible” on the right (score of 10). In this study, the patients will use the VAS to indicate their current pain level at rest and during the most intense exercise,^[16]^ and the VAS score will thus be determined.

The pressure pain threshold (PPT) will also be measured. The PPT refers to the pressure applied when the patient feels pain in a certain area, and it is measured in units of kg/cm^2^. More severe pain is associated with a smaller PPT; conversely, less severe pain is associated with a larger PPT. In this study, a hand-held pain meter will be used to measure the PPT in the painful area of the neck. The patient will be placed in the prone position, and the pain meter will be pressed down onto the skin within the area of measurement. When the patient feels pain, the application of pressure will be stopped, and the screen of the pain meter will show the PPT.

The final primary outcome measure will be the Oswestry disability index (ODI). The ODI consists of 2 parts and a total of 10 evaluation items. The first part assesses low back pain and related symptoms (intensity of pain, concentration, and sleep), and the second part assesses activities of daily living (personal care, heavy lifting, reading, working, driving, and entertainment). The ODI is filled out by the patients according to their own conditions. Each item has a minimum score of 0 and a maximum score of 5, with higher scores indicating more serious dysfunction.

#### 2.7.2 Secondary outcome measures

##### 2.7.2.1 Sagittal balance parameters of the spine and pelvis

The sagittal parameters of the spine will be measured on the full-length lateral spine X-ray film with the patient in the standing position. When the patient is X-rayed, he or she will stand naturally with the knees extended, the eyes looking forward, and upper limbs folded on the chest and lifted up to 45°.

##### 2.7.2.2 Spinal parameters (Fig. 4a)

The following spinal parameters will be measured:

- Lumbar lordosis: the Cobb angle between the L1 endplate tangent and S1 sacral endplate tangent
- Lumbar vertebral angle: the Cobb angle between the anterior edge line of L3 and the perpendicular line of the endplate of the sacrum
- Tilt angle of the first thoracic vertebra line: the Cobb angle between the line from the center of L1 to the midpoint of the sacrum endplate and a horizontal vertical line; when L1 is in front of S1, the value is positive, and when L1 is behind S1, the value is negative.

**Figure 4:**
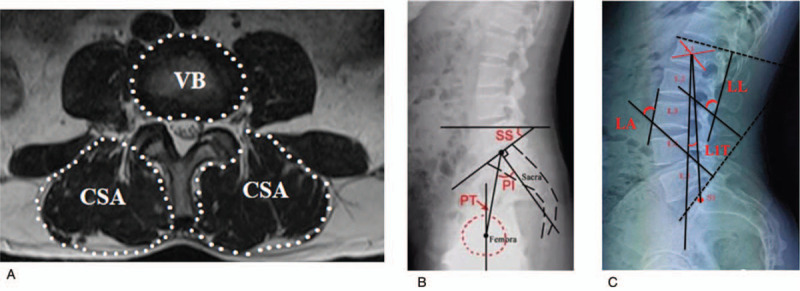
In X-ray and MRI Image, measurements of sagittal parameters of spinopelvic alignment (A and B) and the cross sectional-area (CSA) of the erector spinae (combined liocostalis and longissimus) and the vertebral body size (VB) on the inferior endplate of L1,2,3,4,5 axial (C). LL = lumbar lordosis, LA = lumbar vertebra angle, T1T = Lumbar 1 vertebra tilt angle. PI = pelvic incidence, PT = pelvic tilt, SS = sacral slope.

##### 2.7.2.3 Pelvic parameters (Fig. 4b)

The following pelvic parameters will be measured:

- PI: the angle between the vertical midpoint line of the upper endplate of the sacrum and the midpoint ligature of the sacrum and bilateral femoral heads. The PI is an independent morphological and relatively constant spatial parameter.
- SS: the angle between the upper endplate of the sacrum and the horizontal plane. The SS is a location parameter that varies with the location of the pelvis.
- PT: the angle between the ligature of the upper endplate midpoint of the sacrum and the central line midpoint of the bilateral femoral heads and the vertical plane. The PT is also a position parameter. According to the formula PI = SS + PT, if the SS decreases, the PT increases and vice versa.

##### 2.7.2.4 Lumbar muscularity (Fig. 4c)

MRI examinations will be performed with a Siemens MAGNETOM Skyra 3.0T scanner (Siemens Healthineers, Erlangen, Germany). For the axial view, the scanner will be aligned parallel to the inferior endplate of the vertebral body. The patients will be placed in the supine position with their legs straight and lumbar spine in a neutral posture. Fifteen 4.5-mm-thick transaxial slices will be obtained (3 slices per level) with a 0.1-mm space between each slice. T2-weighted images will then be obtained with the following imaging parameters: 4000-ms repetition time, 123-ms echo time, 438 × 224 matrix, four excitations, and 180-cm field of view.

The following parameters will be measured on the MRI scans: cross-sectional area (CSA) in units of mm^2^, which indicates the size of the multifidus and erector spinae muscles including the portions of fatty change on both sides; the vertebral body (VB) in units of mm^2^, which indicates the size of the bone; and the lumbar muscularity (LM), measured as a percentage and calculated as LM = CSA / VB × 100. The CSA, VB, and LM will be measured on images displayed and analyzed with the PiView digital image viewing software. The CSA will be outlined with a graphic cursor around the paraspinal muscles (multifidus and erector spinae) on both sides, and the VB will be measured at the lower endplate of the L1–5 levels. On both sides, the thoracolumbar fascia will be traced down laterally and anteriorly to the dorsal side of the quadratus lumborum, followed by the posterior surface of the facet and lamina and the lateral margin of the spinous process. The VB will be measured on axial T2-weighted images by constructing polygon points around the outer margins of the bone without the bony spur or disc. The LM will be calculated by dividing the CSA by the VB at the lumbar vertebral level and then multiplying by 100. The average LM will be calculated as follows: LM = (CSA_L1_ + CSA_L2_ + CSA_L3_ + CSA_L4_ + CSA_L5_) / (VB_L1_ + VB_L2_ + VB_L3_ + VB_L4_ + VB_L5_) × 100.

### 2.8 Safety assessment

The project director will supervise the data collection process to assure the safety of the participants. At each visit, the participants will be required to stay in the hospital for 30 minutes after the treatment and will be asked about any adverse effects that may indicate acute adverse reactions during the study period (e.g., lower limb paralysis or cauda equina syndrome). All adverse effects will be recorded at each visit during the treatment period.

### 2.9 Sample size calculation

We estimated the sample size based on the main outcome indicators, and we conducted a randomized trial of Shi-style lumbar spine manipulation combined with Daoyin therapy versus lumbar traction from March to September 2018, with 10 participants in each group. The outcome parameter was the rate of improvement in the NDI (neck disability index) score from baseline to the end of the 8-week treatment. The results showed that the rate of improvement after Shi-based lumbar spine manipulation combined with Daoyin therapy was 92.0%, while that after lumbar traction was 86.6% (*P* = .893) (α = 0.05, test efficiency 1 − β = 0.8, and equivalent standard δ = 0.2).

Because our study protocol will involve the use of a non-inferiority test, according to the number of cases in the clinical non-inferiority and equivalent tests and the equivalent standard,^[17]^ and using counting data (rates) as the outcome indicators, the following formula was used to estimate the number of patients required for this study:

$$n=2\times {\left({\text{U}}_{\alpha }+{\text{U}}_{\beta }\right)}^{2}\times \text{p}(1-\text{p})/\delta^{2}$$

2 × (U_α_+ U_β_)^2^was calculated as 12.365, and N was 29.537 after substituting the above figures, and the sample size of each group was about 30 cases; according to the loss rate of 20%, the sample size of each group was 36 cases, and the ratio of treatment group and control group was 1:1, a total of 72 cases.

### 2.10 Randomization and allocation

After screening, the patients will be randomly divided into 2 groups at a 1:1 ratio. Randomization will be performed using random numbers generated by SPSS version 21.0 (IBM Corp., Armonk, NY, USA). The patients will be randomly divided into a treatment group (n = 36) and control group (n = 36), and the group assignment will be concealed from the researchers by a senior data manager who will not be involved in the study. The group assignments will be sealed in opaque envelopes and be opened by the researchers following acquisition of informed consent and completion of baseline testing.

### 2.11 Blinding

All of the investigators, physicians, nurses, assessors, analysts, and participants will be blinded to the group assignments until the end of the trial, after all statistical analyses are finished. The patients will not be blinded. If any clinically significant adverse event potentially related to the treatment occurs after the first treatment administration, the study physician will re-evaluate the participant and the principle investigator will decide whether the non-blinded procedure is necessary. If non-blinding is required, the allocation information will be provided.

### 2.12 Data collection and monitoring

This is a 24-week clinical trial in which the participants will undergo the research intervention for 4 weeks with a 24-week follow-up. The primary outcome measures will be assessed at 5 time points (at baseline and at weeks 2, 4, 12, and 24), the secondary outcome measures will be assessed at 2 time points (at baseline and at week 24), and safety will be assessed at 6 time points (at baseline and at weeks 1, 2, 4, 12, and 24). All results will be independently documented by 2 investigators using EpiData version 3.1 (EpiData Association, Odense, Denmark), and differences will be resolved through discussion with a third investigator. The Longhua Hospital Affiliated to Shanghai University of Traditional Chinese Medicine will be responsible for monitoring and quality control.

### 2.13 Statistical analyses

Trained statisticians will use the intentionality approach for validity assessment and safety analysis. The final outcome observation method will be applied to deal with missing values. All statistical analyses will be performed using SPSS version 22.0 (IBM Corp.). Before randomization, the patients baseline characteristics will be collected as descriptive statistics, including sex, age, body mass index, duration of symptoms, and degree of LDH. The data analysis of the primary outcome will be based on the per-protocol population as a supportive analysis. The mean, standard deviation, median, quartiles, and interquartile ranges will be calculated for continuous variables, and the frequency will be calculated for categorical variables. Continuous variables with a normal distribution will be calculated by the independent-samples Student *t* test and presented as mean with standard deviation; otherwise, the data will be analyzed using non-parametric tests and expressed as median with range.

Categorical variables will be expressed as number (%) and analyzed by the χ^2^ test or Fisher exact test. Repeated-measures analysis of variance will be used to analyze the changes in the VAS score, PPT, and ODI score at different time points (baseline and weeks 1, 2, 4, 12, and 24). An independent-samples *t* test will be used to analyze the changes in lumbar lordosis, lumbar vertebral angle, tilt angle of the first thoracic vertebra line, PI, PT, SS, and LM at baseline and 24 weeks. A *P* value of <.05 will be defined as statistically significant, and two-sided 90% confidence intervals will be calculated. Missing data will be input with the last observed response carried forward for all measures using the “last-value-carried-forward” principle.

### 2.14 Monitoring

Two supervisors will be sent to the study site twice a month to guarantee the quality of the whole trial. These supervisors will audit the clinical trial, including adverse events, inclusion and exclusion criteria, participant compliance with the clinical trial process, and completion of the case report forms. Standard operating procedures will be invariably followed. Drop-outs, withdrawals, and any noncompliance will be recorded in detail by the inspectors throughout the treatment and follow-up periods. This process will be independent from the investigators and the sponsors.

## 3 Discussion

Manipulation and guidance (Daoyin) are both traditional Chinese medicine treatment methods. Manipulation is a means for the purpose of treatment of using hands or other parts of the body, according to various specific skills, to manipulate on patients; Daoyin belongs to the category of exercise, and it is to reach the goal of treating diseases and keeping health by combining breathing regulation and limb movement. Manipulation is dominated by doctors, who purposefully choose different manipulations to apply to patients according to their needs. The Daoyin is dominated by the patients themselves. The patient chooses the appropriate Daoyin according to his or her physical condition and disease state. Shi-style spinal balance manipulations can restore disordered small joints, slightly pulling open the intervertebral foramen and applying traction to the sciatic nerve, which helps to relieve the adhesion of the lateral nerve root. The comprehensive effect of converting mechanical energy into heat energy by means of manipulation can promote blood circulation and thus move blood stagnation. It can not only accelerate the absorption of edema and relieve local aseptic inflammation, but it can also increase local blood flow, improve the nutrition of diseased tissues, and promote the repair of lesions.^[18]^ It also has the effect of relieving pain. By applying different strengths and manipulations to different muscle layers that need to be treated, the local pain threshold can be increased to alleviate the pain. Muscle and Bone Guidance, a type of Daoyin can adjust the body, relax the muscles, strengthen the bones, and prevent muscle atrophy; it can also improve the flexibility of the dorsolumbar region, adjust the tension of local muscles, improve the coordination of the dorsolumbar flexor and extensor muscles, and increase the stability of the spine, thus preventing recurrence of LDH.

Research has shown that Tuina manipulation combined with Daoyin is more effective than regular manipulation alone.^[19]^ When performing Shi-style spinal balance manipulation, the patient is mainly static, and the treatment is focused on bone reinforcement and consolidation to restore the patients endogenous stability. However, when performing Muscle and Bone Guiding exercise, movement is the main treatment method, focusing on soothing the tendons and strengthening the muscles and aiming to restore the patients exogenous stability. Tuina manipulation and the manipulation and Daoyin play an important role in nonsurgical therapy, which can improve the root symptoms caused by LDH. Additionally, some clinical studies have shown no significant difference the long-term outcome of surgical and nonsurgical treatment for LDH.^[20]^ However, because of the variety of Tuina manipulation and guidance (Daoyin) techniques and the lack of standards and norms, deviation of clinical efficacy and even aggravation of the disease can readily occur. Although the diagnosis and treatment standards of surgical therapy and drug therapy are relatively clear and unified and exhibit stable and accurate curative effects, surgical therapy is associated with a certain degree of trauma and high cost; it can also lead to postoperative complications, the treatment of which is difficult for some patients to afford. At the same time, long-term use of nonsteroidal drugs can easily cause gastric mucosal damage or renal metabolic disorders.^[21,22]^ Therefore, we plan to conduct this multicenter, prospective randomized controlled clinical study to compare the effectiveness of Shi-style spinal balance manipulation combined with bone guidance (Daoyin) versus mechanical pelvic traction and explore the effect of this treatment on the sagittal balance of the spine and structural parameters of the lumbar spine in elderly patients with complicated LDH. The results of this study will clarify the effectiveness and feasibility of Shi-style spinal balance manipulation combined with bone guidance (Daoyin) and indicate whether it can be a potential conservative treatment option for LDH.

## Acknowledgments

We thank Angela Morben, DVM, ELS, from Liwen Bianji, Edanz Editing China (www.liwenbianji.cn/ac), for editing the English text of a draft of this manuscript.

## Author contributions

Xing Ding, jinhai Xu, Jinze Wu are co-first authors of this manuscript, contributing equally to the design, conduct of the trials, and drafting of the manuscript. All authors participated in the design of the study and performed the trial. Jie Ye and Wen Mo are the co-corresponding authors of this manuscript, contributing equally to the supervision and coordination of the clinical trial. All authors read and approved the final manuscript.

**Conceptualization:** Xing Ding, Jinhai Xu, Jinze Wu, Xiaoning Zhou, Kun Jin, Ming Yan, Xuequn Wu, Jie Ye, Wen Mo.

**Data curation:** Xing Ding, Jinhai Xu, Jinze Wu, Kun Jin, Jie Ye, Wen Mo.

**Formal analysis:** Xing Ding, Jinhai Xu, Jinze Wu, Xiaoning Zhou, Kun Jin, Jie Ye, Wen Mo.

**Funding acquisition:** Xing Ding, Jinhai Xu, Ming Yan.

**Investigation:** Xing Ding, Jinze Wu, Xiaoning Zhou, Ming Yan.

**Methodology:** Jinze Wu, Kun Jin, Ming Yan, Junming Ma, Xuequn Wu.

**Project administration:** Junming Ma, Xuequn Wu, Wen Mo.

**Resources:** Xing Ding, Xiaoning Zhou, Junming Ma, Xuequn Wu, Wen Mo.

**Software:** Xing Ding, Jinhai Xu, Jinze Wu, Kun Jin, Junming Ma, Jie Ye, Wen Mo.

**Supervision:** Jinhai Xu, Jinze Wu, Kun Jin, Ming Yan, Junming Ma, Jie Ye.

**Validation:** Jinhai Xu, Ming Yan, Xuequn Wu, Jie Ye, Wen Mo.

**Visualization:** Xiaoning Zhou, Kun Jin, Wen Mo.

**Writing – original draft:** Jinze Wu, Xiaoning Zhou, Ming Yan, Jie Ye, Wen Mo.

**Writing – review & editing:** Kun Jin, Wen Mo.
